# Mapping Influenza-Induced Posttranslational Modifications on Histones from CD8+ T Cells

**DOI:** 10.3390/v12121409

**Published:** 2020-12-08

**Authors:** Svetlana Rezinciuc, Zhixin Tian, Si Wu, Shawna Hengel, Ljiljana Pasa-Tolic, Heather S. Smallwood

**Affiliations:** 1 Department of Pediatrics, University of Tennessee Health Science Center, Memphis, TN 38163, USA; srezinci@uthsc.edu; 2 Environmental Molecular Sciences Laboratory, Pacific Northwest National Laboratory, Richland, WA 99354, USA; zhixintian@tongji.edu.cn (Z.T.); si.wu@ou.edu (S.W.); shengel@seagen.com (S.H.); Ljiljana.PasaTolic@pnnl.gov (L.P.-T.); 3 Children’s Foundation Research Institute, Memphis, TN 38105, USA

**Keywords:** epigenetic, histone, posttranslational modifications, T cells, influenza, top-down, mass spectrometry

## Abstract

T cell function is determined by transcriptional networks that are regulated by epigenetic programming via posttranslational modifications (PTMs) to histone proteins and DNA. Bottom-up mass spectrometry (MS) can identify histone PTMs, whereas intact protein analysis by MS can detect species missed by bottom-up approaches. We used a novel approach of online two-dimensional liquid chromatography-tandem MS with high-resolution reversed-phase liquid chromatography (RPLC), alternating electron transfer dissociation (ETD) and collision-induced dissociation (CID) on precursor ions to maximize fragmentation of uniquely modified species. The first online RPLC separation sorted histone families, then RPLC or weak cation exchange hydrophilic interaction liquid chromatography (WCX-HILIC) separated species heavily clad in PTMs. Tentative identifications were assigned by matching proteoform masses to predicted theoretical masses that were verified with tandem MS. We used this innovative approach for histone-intact protein PTM mapping (HiPTMap) to identify and quantify proteoforms purified from CD8 T cells after in vivo influenza infection. Activation significantly altered PTMs following influenza infection, histone maps changed as T cells migrated to the site of infection, and T cells responding to secondary infections had significantly more transcription enhancing modifications. Thus, HiPTMap identified and quantified proteoforms and determined changes in CD8 T cell histone PTMs over the course of infection.

## 1. Introduction

Clearance of viral infection depends upon a well-orchestrated immune response and requires precise control of the immediate effector T cell response as well as the formation and maintenance of the memory T cell population. Activation of naïve T cells (Tn) initiates an autonomous program of differentiation and the acquisition of effector functions, including pro-inflammatory cytokine and cytolytic effector molecule production [1,2,3,4,5]. Effector CD8 T cells (Teff) modulate their transcriptional programs as they adapt to activation stimuli and immune resolution, which influences their differentiation status and function. Thus, in response to dynamic environmental conditions, naïve cells alter their signaling cascades and pathways, leading to the induction of cytokine production and robust cytolytic activity. After the resolution of viral infection, Teff populations contract, and a small population of pathogen-specific long-lived memory cells remain. Memory CD8 T cells (Tm) are imprinted during the primary infection, and their robust recall response dictates the efficiency of the secondary immune response. Thus, virus-specific Tm are preprogrammed to respond rapidly to subsequent infections without further differentiation [5,6,7,8,9,10,11]. It has long been appreciated that Tn, Tm, and Teff have distinct phenotypes and functions. Yet, the origin of Tm remains unclear as there is evidence for both their development early in activation as well as from fully differentiated Teff. It is certain that epigenetics plays a role in both Tm and Teff generation. However, we are just beginning to understand the underlying epigenetic mechanisms that control the maintenance of these subsets and their unique responses to infection.

During T cell activation, in addition to mobilizing signal transduction cascades and transcription factors to drive the production of proteins necessary for effector functions, enzymes that control the epigenetic imprinting of CD8 T cells are upregulated [12]. These epigenetic imprints are posttranslational modifications (PTMs) to histones and DNA that form heritable changes without altering primary DNA sequences [3,13,14,15,16,17,18]. In the case of histones, this produces unique proteoforms which are the different molecular forms created by PTMs to the protein product of each of the canonical histone genes (H1, H2A, H2B, H3, and H4) [19]. As they differentiate, T cells change histone PTMs at gene loci associated with effector function [3,13,14,15,16,17]. Thus, combinations of histone PTMs at discreet genomic locations can indicate transcription levels and regulate cell-type-specific gene expression patterns [20,21]. The amino-terminal histone tails protruding from the nucleosome subunits are the main PTMs sites for acetylation, methylation, and phosphorylation that directly alter DNA accessibility or act indirectly via binding or chaperoning other proteins [22,23]. Acetylation of lysine residues on histones reduces their positive charge, weakening the electrostatic interaction with DNA. This “permissive” state allows transcription factors to access genes in this region. Conversely, when histone deacetylases remove these PTMs, the structure condenses, thereby restricting access to the region [24]. Thus, maintenance of PTMs by these enzymes play central roles in controlling T cell development [25,26,27], regulatory function maintenance [28], CD8 T cell proliferation [29], and effector functions, including the anti-viral response of antigen-specific CD8 T cells [30,31]. In contrast to the on/off effects of acetylation, the effects of histone methylation are context-dependent. For example, trimethylation of histone 3 or 4 at lysine 4 or 9, respectively, is transcriptionally permissive, while the location and degree of methylation of lysines 27 and 9 on H3 within gene loci typically correlates with transcriptional repression [21,32,33,34,35,36,37,38,39,40,41]. This context dependency comes into play when both marks are present (bivalent), resulting in genes that are poised for expression pending the removal of the repressive modification. Bivalent marks on H3 are thought to contribute to rapid T cell differentiation [42,43,44]. Mapping gene expression patterns at loci associated with H3K4me3 and H3K27me3 from Tn, Teff, and Tm revealed the expression of T cell lineage-defining genes correlated with phenotypic and functional differences between virus-specific CD8 T cell subsets [3]. Following influenza infection, the transition from naïve to effector T cell was characterized by the loss of repressive H3K27me3 at specific bivalent loci and provided a mechanistic basis for the coordinate regulation of transcription during differentiation.

H3 trimethylations are the most widely studied histone PTMs. However, the histone octamer contains two H2A and H2B dimers as well as a tetramer of H3 and H4 proteins. Given the emerging role of epigenetics in CD8 T cell regulation, deciphering the histone code is of immediate importance. Analyzing intact histones by top-down mass spectrometry (MS) is a major advantage when tackling the histone code because combinatorial patterns of modifications on a single histone molecule can be identified [45]. Here, we used two-dimensional liquid chromatography-MS/MS (2D LC–MS/MS) for histone-intact protein PTMs mapping (HiPTMap) of H2A, H2B, H3, and H4 purified from CD8 T cells following influenza infection. This approach allowed us to assess the dynamics of the PTM landscape in naïve and activated T cells in the primary infection, map PTMs as effector T cells migrated to the site of infection, and compare Tm with Teff from the primary and secondary responses. We identified 225 unique PTMs on histone proteoforms from the spleen, bronchial lavage fluid (BAL) and lungs, found distinct PTM combinations based on T cell subsets and found a significant increase in enhancer PTMs in Teff from the secondary response. HiPTMap is particularly valuable for advancing our understanding of T cell biology by providing relative quantification of PTMs in different cell states with minimal histone protein. Furthermore, these results demonstrate this approach and technological advancements in MS are poised to increase the identification of novel modification locations and combinations that will generate a complete map of the histone code in distinct T cell populations. Histone PTMs identified in this study may also shed light on new regulatory mechanisms and allow for the detection of new loci relevant to T cell function and fate. The extent to which histone modifications direct T cell differentiation and fate remain unclear. Identifying the histone code for T cells at different locations and times following influenza infection may elucidate functional mechanisms in chromatin regulation in CD8 T cells and provide new insights into epigenetic maintenance of T cell phenotypes.

## 2. Materials and Methods

### 2.1. Infections and T Cell Extraction

Female C57BL/6 mice (The Jackson Laboratory, Bar Harbor, ME, USA) were infected for 9 days for primary and ≥30 days for memory or secondary challenge. Mice were intranasally infected with influenza A virus strain A/X-31(H3N2) (X31) at EID_50_ = 10^6^. For the challenge, at least 30 days prior to X31 infection, mice received an intraperitoneal injection of A/Puerto Rico/8/1934 at EID_50_ = 10^8^. Mice were sacrificed at specific times after X31 infection (9 days for Teff^1^, 7 days for Teff^2^, and 30 days for Tm). In each experiment, we used 8–10 mice for Teff^1^, 20–40 for Tm, and 5–6 mice for Teff^2^ per group. Spleens were collected, and CD8 cells separated [5,10,46] using B220, MHCII, CD11b, and DX5 (NK) antibody cocktail (Miltenyi Biotec, Auburn, CA, USA). Fluorescence-activated cell sorting (FACS) was used to isolate CD8 T cells. Activated splenic T cells were selected based on CD8^hi^ CD44^hi^ CD43^hi^ CD25^hi^, and naïve cells were selected based on CD8^hi^ CD44^low^ CD43^low^ CD25^low^. T cells from BAL were selected based on CD8^+^ CD44^hi^ CD43^hi+^ CD25^+^, Lung CD8^+^ CD44^hi^ CD43^hi^ CD25^mid-hi^. The use of CD25 with CD43 was recently shown to allow isolation of influenza-specific CD8 from the BAL, lung and spleen [10]. The pellets were resuspended in ammonium bicarbonate with protease inhibitor cocktail (Roche, Indianapolis, IN, USA). Histones were purified using a histone purification kit (Active Motif, Carlsbad, CA, USA) for purifying core histones while preserving modification states. Columns were poured for each group and kept constant. We followed the manufacturer’s protocol for gravity flow separation of H2A, H2B, H3 and H4 core histones. Protein was purified from each group, and 9 µg protein per run was used for MS analysis. For relative comparisons (e.g., naïve vs. active or active spleen vs. BAL vs. lung), mice and samples were treated as above, except histones were pooled for relative quantification (10 mice). Based on these and other preliminary experiments (DNS), we estimate 2–5, 10–15, or 1–2 mice per group are required for the primary, memory, and secondary response groups, respectively, for adequate coverage of all core histones with this method.

### 2.2. Mass Spectra Acquisition

We previously developed an optimized MS workflow for HiPTMap and provided a detailed MS configuration and separation strategy [47]. Briefly, purified histones (7.5–10 μg) were separated in the first dimension using a Jupiter C5 column (5 μm particles, 300 Å pore size, 600 mm × 200 μm i.d.) (Phenomenex, Torrance, CA, USA). Two Model 100 DM 10,000 psi syringe pumps (ISCO, Lincoln, NE, USA) were used to maintain constant pressure (4000 psi) during separation. A gradient was generated by adding mobile phase B to a stirred mixer of mobile phase A (2.5 mL volume at 100% “A” at time zero). Mobile phase A was 20% acetonitrile (CAN) aqueous solution with 5% isopropyl alcohol (IPA) and 0.6% formic acid (FA). Mobile phase B was 45% ACN, 45% IPA, and 0.6% FA. The appropriate split flow rate was controlled by the combination of a packed column together with 15 μm i.d. capillary, with an approximate flow of 10 µL/min. RPLC separation was directly coupled with the mass spectrometer for comparative analyses (details provided below). Alternatively, we used a SPECTRA100 UV detector (Thermo Separation Products, Waltham, MA, USA) to monitor protein elution online at 214 nm, and fractions were collected using a Cheminert column selector system (VICI, Houston, TX, USA).

Each histone family fraction was further separated in the second dimension by weak cation exchange hydrophilic interaction liquid chromatography (WCX-HILIC) using a 50 cm × 100 μm i.d. column prepared with PolyCAT A (5 μm particles, 1000 Å pore size) (PloyLC, Columbia, MD, USA). We used a 70% ACN aqueous solution with 1.0% FA for mobile phase A (2°A) and 70% ACN and 8% FA for mobile phase B (2°B) for the gradient in the second-dimension separation. To increase the throughput of the second dimension, we employed a ten-port nanovolume injection valve (VICI) to house two capillary columns, enabling separation and concurrent loading/equilibration between the two columns. Each isolated core histone fraction was loaded onto a 150 μm i.d. × 5 cm solid-phase extraction column with HILIC stationary phase described above. After loading each fraction in mobile phase 2°A, mobile phase 2°B was added to the mixing vessel to separate the proteoforms of each core histone for more effective MS characterization. We acquired spectra using electrospray ionization (ESI) high-resolution MS and MS/MS acquisitions in an LTQ Orbitrap Velos (Thermo Fisher Scientific, Waltham, MA, USA). ESI voltage was applied by connecting the end of the LC column to a capillary emitter with a polyether ether ketone (PEEK) union while voltage was applied [47]. Acquisitions with the Orbitrap had nominal resolving power of 60,000 (m/z = 400). FTMS MS and MSn gain control were set at 1E6 and 3E5, respectively, with three micro scans each. Precursor ion fragments were isolated with a 1.5 m/z window. The collision-induced dissociation (CID; normalized collision energy 35%, 30 ms) and electron transfer dissociation (ETD; reaction time 25 ms) for the same precursor ion were alternated, and exclusion duration of 900 s and an exclusion list size of 150 were applied. MS/MS was only performed on species with charge states greater than four.

### 2.3. Data Analysis

Proteoforms, as well as PTMs, were identified by searching raw data against a mouse-specific annotated database, mouse op down database (1260319 basic sequences, 5,097,711 protein forms), using ProSightPC 2.0 software (Thermo Scientific) using the Xtract algorithm and top-down (MS2) default settings. The analysis was performed in single-protein search mode with dynamic modifications used for the identification of PTMs, including acetylation, mono-, di-, and trimethylation and phosphorylation with a mass tolerance of 10 ppm for precursor and 10 ppm for fragments using Δm mode (this function allows identification of proteoforms with PTMs not included in the annotated proteoform database). The minimum signal-to-noise (S/N), minimum reliability (RL), maximum charge and maximum mass were set to 1.0, 0.9, 40, and 25 kDa, respectively. Individual spectra were searched in absolute mass mode if a minimum of six fragments and a minimum intact mass of 5000 Da were observed, and the fragment mass tolerance was set at 10 ppm. Due to limitations in the ProSightPC search tool (i.e., restricted to previously annotated PTMs), a manual analysis was performed in the few cases ProSightPC could not identify species of import between biological comparisons. Histone identifications were filtered by requiring the “number of best hits” to be one (globally unique ID). The false discovery rate (FDR) was evaluated using a reversed database search with the same filtering criteria, where FDR = 100*Nreverse/Nforward. A P score cutoff of 0.00025 was chosen with FDR < 1%. MS Access was used as a platform to align and normalize individual histones from a 2D display for further inference of LC–MS datasets of histone proteoforms generated from T cell subsets. Proteoforms obtained from the search were grouped in MS Access to give the final globally unique isoforms with normalized intensities across all the identified isoforms for quantitation. Histone PTM analysis is a computationally challenging process we refer readers to for details and tips to analyzing these data [47,48,49,50,51].

## 3. Results

### 3.1. Naïve Versus Activated T Cell Histone Modifications

**Figure 1:**
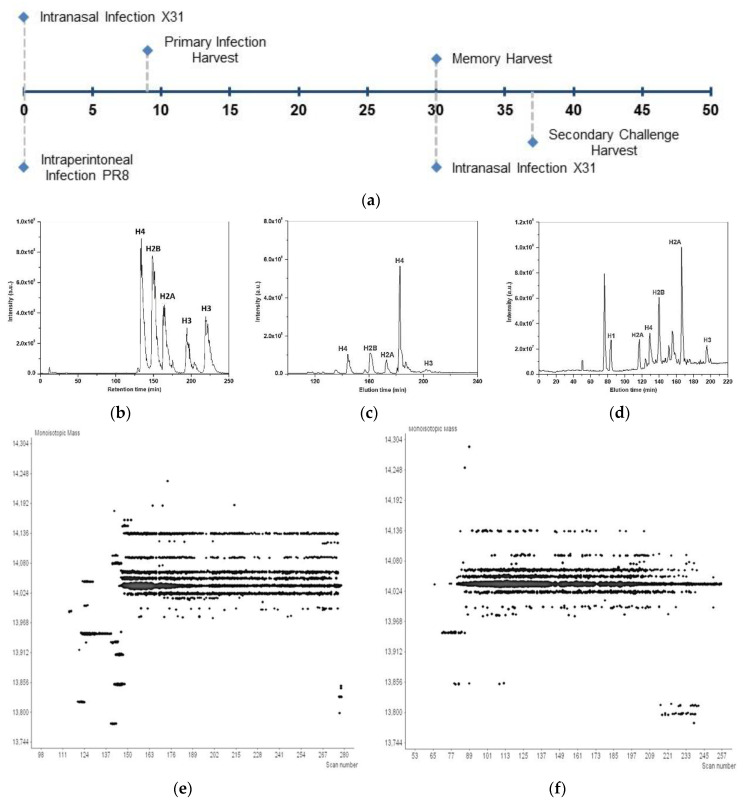
Representative mass spectral data of core histones from CD8 T cells. (**a**) Female C57BL/6 mice were used with 2–5, 10–15, or 1–2 mice per primary, memory, and secondary infection, respectively. Mice were intranasally infected with influenza A virus strain A/X-31(H3N2) (X31) at EID_50_ of 10^6^ and sacrificed on day 9 for the primary response or after 30 days for the memory response. For the secondary response, mice received intraperitoneal injections of PR8 at EID_50_ of 10^8^ and, after 30 days, were challenged with X31 as described above. These mice were sacrificed 7 days after the heterologous challenge. In the primary infection, bronchial lavage fluid (BAL) was taken from mice, lungs were profused then harvested, and spleens were harvested. Prior to sorting, crude cell preparations from mice were grouped, and contaminated immune cells were reduced by negative depletion. Enriched CD8 T cells were stained for surface markers and sorted by FACS. Naïve cells were selected based on CD8hi CD44low CD43low CD25low from the spleen in the primary. Activated T cells from the spleen were selected based on CD8hi CD44hi CD43hi CD25hi from the primary and secondary responses. Enriched Tm from the spleen were selected based on CD8hi CD44+ CD43+. T cells from BAL were selected based on CD8+ CD44hi CD43hi+ CD25+ and CD8+ CD44hi CD43hi CD25mid-hi from the lung. Histones were purified from T cell populations using a histone purification kit (Active Motif, Carlsbad, CA, USA) for purifying core histones while preserving modification states. Each population had a dedicated column, and we followed the manufacturer’s protocol for gravity flow separation of H2A, H2B, H3 and H4 core histones. 9 µg of histone protein was used from each experimental replicate for MS analysis. For relative comparisons (e.g., naïve vs. active or active spleen vs. BAL vs. lung), mice and samples were treated as above except histones were pooled for relative quantification (10 mice each); (**b**) Histones were purified and subjected to RPLC–MS/MS analysis on an Orbitrap Velos mass spectrometer. Mass spectra were acquired with high-resolution to ensure isotopic resolution for all detected protein species. Representative data are provided for the total ion chromatograms of histones from HeLa cell standards (**b**), naïve CD8+ T cells (**c**) and activated CD8 T cells (**d**); (**e**,**f**) Two-dimensional displays depicting resolved intact protein masses of H2A from two independent experiments; species were detected using reversed-phase separation for activated T cells.

We previously used 2D LC–MS/MS) for HiPTMap of proteins purified from HeLa cells and demonstrated the high sensitivity and comprehensive characterization of histone PTMs [47]. Here, using our established methods [3,5,52], female C57BL/6 mice were intranasally infected with influenza strain X31, spleens harvested nine days later, and T cells isolated by FACS (Figure 1a). Histones were purified from T cell lysates and subjected to LC-MSanalysis. We used high-resolution reversed-phase liquid chromatography with tandem mass spectrometry (RPLC–MS/MS) on an Orbitrap Velos with alternating CID and ETD. We applied this MS method for HiPTMap of modifications that formed in vivo following intranasal infection with influenza virus. Both MS and MS/MS datasets were acquired with high-resolution and mass accuracy (intact protein below 1 Da and fragment ions below 10 ppm). All core histones were loaded onto the columnand separated into individual families H4, H2B, H2A, and H3 that eluted in increasing order of molecular weight, which roughly correlates with increasing hydrophobicity (i.e., 11,352.5, 13,757.1, 14,019.9, and 15,350.8 Da, respectively) (Figure 1a–c). Proteoforms within each histone core family eluted together, except for H3. With reversed-phase separation, elution time is primarilyinfluenced by hydrophobicity. Thus, H3 eluted in two distinct peaks; the second peak contained H3 species with up to three additional methylations that increase their hydrophobicity (Figure 1a–c). This clear separation of histone core family members allows for facile separation, fragmentation, and downstream HiPTMap.

The high degree of similarity between various histone proteoform masses and the considerable difference in the amounts of each core histone (Figure 1b,c)necessitated a combination of high-resolution protein separation and high-performance MS analysis for the identification of individual histone proteoforms. Raw data were searched against mouse-specific annotated data to map top-down fragmentation spectral data to associated sequences for the identification of PTMs, including acetylation, mono-, di-, and trimethylation and phosphorylation. Supplemental File S1 lists the identified proteoforms, exact masses, and significance for identifications from the naïve and activated T cells (tabs A and B, respectively). We identified 166 unique histone proteoforms from naïve and activated CD8 T cells isolated from the spleens of mice infected with influenza for 9 days (Table 1). We validated the identity of these proteoforms using online nanoflow WCX-HILIC LC–MS/MS (data not shown). WCX-HILIC MS/MS uses specialized separation for the characterization of complex mixtures of hypermodified combinatorial proteins. However, WCX-HILIC only marginally increased the number of identified proteoforms in the naïve T cells, likely due to limited amount of material available for analysis. To reduce sample requirements, we continued with the RPLC–MS/MS-CID/ETD approach for HiPTMap and comparative analyses.

**Figure 2:**
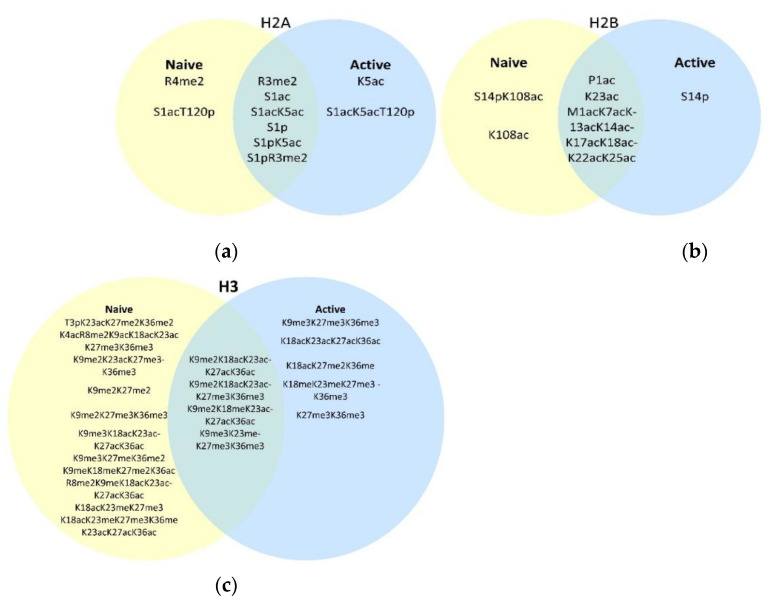

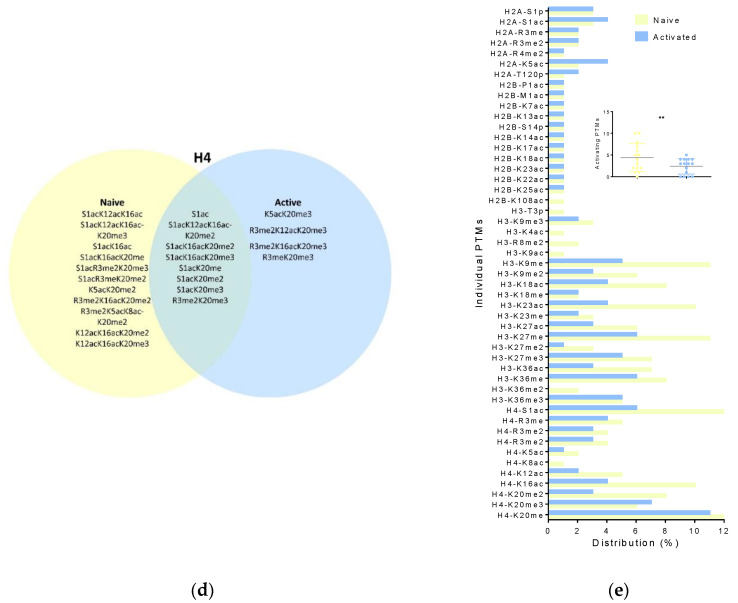
Histone-intact protein PTM mapping (HiPTMap) of naïve and activated CD8 T cells. (**a**–**d**) Histones purified from naïve (CD8^hi^ CD44^low^ CD43^low^ CD25^low^) or activated T cells (CD8^hi^ CD44^hi^ CD43^hi^ CD25^hi^) purified by FACS of splenocytes from mice 9 days after intranasal infection with influenza and subjected to RPLC–MS/MS using Orbitrap Velos with alternative collision-induced dissociation (CID)/alternating electron transfer dissociation (ETD). Raw datasets were searched against the mouse top-down database. PTMs including acetylation, mono-, di-, and tri-methylation and phosphorylation were detected; (**e**) the individual modifications from each proteoform were combined to yield the sum of each PTM per core histone. A Wilcoxon matched-pairs signed-rank test was performed (** *p* < 0.0001) with pairing evaluated by Spearman correlation (** *p* < 0.0001). Inset: the number of enhancer PTMs (H4-K8ac, H3-K36me2, H3-K9ac, H2B-K12ac, H4-K5ac, H3-K36me3, H4-K12ac, H4-R3me2, H3-K36ac, H3-K27ac, H2A-S1p, H4-K16ac, H3-K23ac, H3-K18ac, and H2A-K5ac) were compared for the activated and naïve with paired *t*-test (*p* = 0.0046).

Histones from naïve T cells had more total unique modified species detected and identified than those from activated T cells (Table 1). H2A was the only core histone with more unique modified sequences from activated T cells, and six out of nine contained K5ac. This enhancer mark was predominantly associated with the activated T cells (Table S1). K5ac was distributed more frequently among PTM combinations after activation and with increased frequency. This trend remained true when K5ac was accompanied by serine 1 acetylation and/or threonine 120 phosphorylation (i.e., five found in activated T cells, one in naïve) (Table S1). R3Me2 and R4me2 were reduced or not detected after activation, but their function is currently unclear. Core histones from H2B, H3, and H4 had more unique modified species detected in histones purified from naïve T cells (Table 1). Lysine 108 acetylation was specific to H2B from naïve T cells (Table S1). The peak intensity of H3 was lower than that of other core histones (Figure 2), resulting in low overall intensities for H3 proteoforms from both naïve and activated T cells. However, H3 and H4 were generally more heavily modified, and these modifications were often associated with each other (Figure 2c,d and Table S1). For example, lysine 9 through 36 on H3 were often acetylated and/or methylated in groups as were those on H4, whereas serine 1 acetylation was often accompanied by acetylation at positions 12 and 16 and methylation at position 20 (Figure 2d and Table S1). In general, H4 had a relatively high level of triple and quadruple modifications that were distributed on a few amino acids (Figure 2d and Table S1) and was high in abundance for both T cell subsets (Table S2). One modification on H4 that was detected on twice as many unique sequences in the naïve subset was serine 1 acetylation (Table S1), and these proteoforms had higher relative intensities when compared to activated T cells (Table S2). In fact, several of the H4 S1ac proteoforms were not detected in activated T cells (e.g., S1acK12acK16ac, S1acK12acK16acK20me3, S1acK16ac, S1acK16acK20me, S1acR3me2K20me3, and S1acR3meK20me2). S1ac and R3me appear to be mutually exclusive, and the latter was more evenly distributed across the T cell states (Figure 2d). This is similar to H3, where arginine methylation restricts phosphorylation of S1. Given that the proteoforms containing S1ac account for 38% of total histone proteoforms in the naïve T cells and only 13% in activated T cells, this mark may be of importance prior to activation (Table S2). These findings indicate individual and combinations of PTMs are distinct in naïve versus activated T cells from primary influenza infection.

To better compare histone proteoforms from naïve and activated T cells, we normalized one representative dataset with the total histone proteoform intensities within the naïve and activated T cells (Table S2). H2A accounted for the largest percentage of histone cores in activated T cells. We found acetylation of serine 1 on H2A (S1ac) was increased in relative intensity (i.e., 23.4% versus 6.5%) and in the number of unique sequences identified in activated versus naïve T cells (Tables S1 and S2). The relative abundance of proteoforms with phosphorylated serine 1 was also higher in activated versus naïve T cells (i.e., 21.3% versus 7.0%) (Table S2). Overall, H2B showed very few differences in counts or relative abundance for naïve and activated T cell subsets (Tables S1 and S2). However, both the fragment numbers and peak intensity of P1ac were higher for H2B from activated T cells (Figure 2b and Table S1). Interestingly, we identified four times more H2BS14p fragment ions from activated T cells than from naïve (i.e., 24 to 6). The relative abundance of H3 was the same irrespective of activation (Table S2). H4 was the dominant species in the naïve T cells. Acetylation of serine 1 accounted for 38.1% of the proteoforms detected in naïve and only 13.6% in activated T cells. Indeed, when we compared acetylation of the first twenty amino acids on H4, it encompassed 50.7% of all species from naïve T cells versus 20.5% from activated T cells. Apart from these exceptions, the majority of modified species we identified were present in histones from naïve and activated T cells to a similar extent (Tables S1 and S2).

To determine if individual modifications changed following activation, compared the total number of times each PTM was identified on the proteoforms of each core histone family member (Figure 2e). The distribution and median of PTMs per core histones from naïve and activated T cells were significantly different (Figure 2e). Six modifications were detected in naïve but not in activated T cells: H2BK108ac H3T3p, H3K4ac, H3R8me2, H3K9ac, and H4K8ac (Figure 2b,c and Table S1). Of these, H3K9ac and H4K8ac are associated with active gene expression, while H3R8me2 is associated with repression [53,54,55,56]. H3 lysine 27 was also acetylated; this activation signal was detected twice as many times for naïve T cells than for activated (i.e., 108 versus 51 fragments corresponding to 6 versus 3 unique proteoforms). Overall, we detected significantly more known enhancers of gene expression for naïve T cells (Figure 2e inset). There was not a significant difference in total repressive marks between naïve and activated T cells (DNS). However, we found a reduction in H3K27me2 and H3K27me3 repressive marks in activated T cells (i.e., 102 versus 68 fragments) (Figure 2e). This was consistent with previous findings [3], and this trend also occurred for dimethylations and monomethylation of lysine 27 (Table S1 and File S1A,B). Given H3K27me3 is associated with repression of transcription of genes in loci related to effector function, these data are consistent with the decommissioning of these repressive marks following T cell activation.

### 3.2. Differences in Effector T Cell Histone Modification from the Spleen, Lung, and Bronchial Lavage

**Figure 3:**
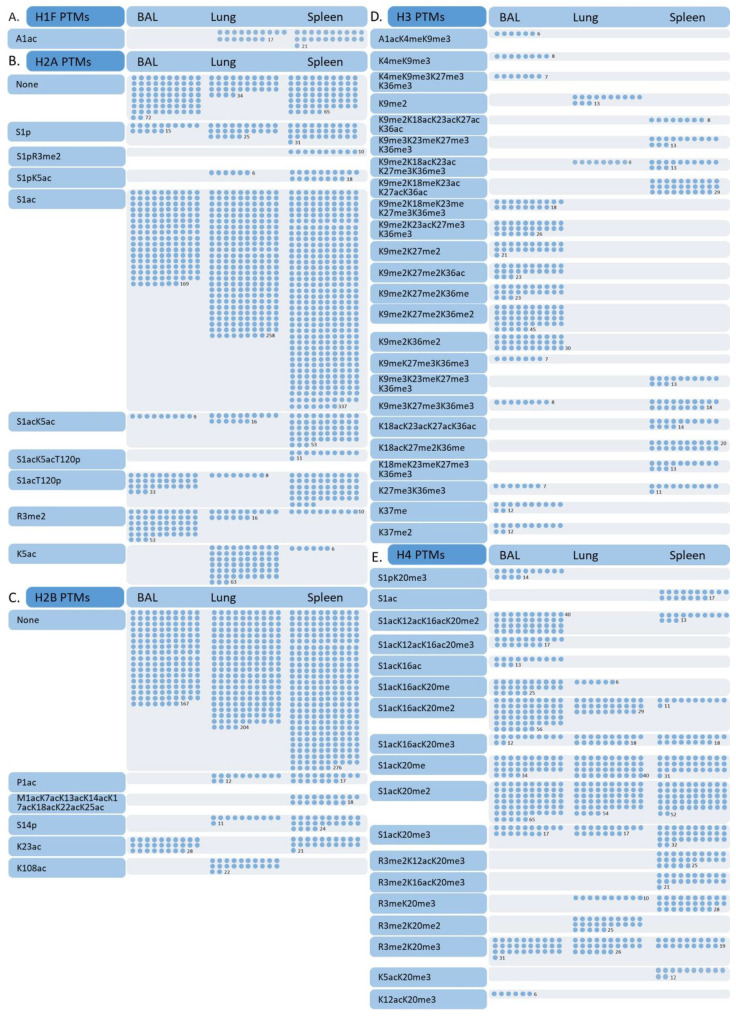
Distribution of modified species identified in histones purified from activated CD8 T cells isolated from BAL, lung, and spleen after influenza infection. Activated T cells were isolated from BAL, lung, and spleen 9 days after influenza infection using FACS (CD8^hi^ CD44^hi^ CD43^hi^ CD25^hi^). Histones were purified from each sample, subjected to activated T cells purified by FACS of splenocytes from mice 9 days after intranasal infection with influenza, and subjected to reversed-phase liquid chromatography (RPLC)–MS/MS-CID/ETD. Fragment ions identified per modified species (●) are indicated in score charts for H1F (**A**), H2A (**B**), H2B (**C**), H3 (**D**), and H4 (**E**).

Influenza infection activates CD8 T cells to expand and differentiate into Teff that migrate to the respiratory tract and contribute to viral clearance [4]. We sought to determine if the relative abundance of histone modifications changed as Teff migrated to the site of infection. We compared Teff from the spleen, lung, and BAL removed from the same mice nine days following influenza infection. Identical proteoforms were grouped, and intensities normalized across species for relative quantification. The observed masses, number of fragments per proteoform, PTMs per proteoform, associated *p* values and normalized intensities values are provided in Supplemental File S2. We found acetylation of alanine one on H1F was similar in Teff from the lung and spleen (Figure 3A). In contrast, we found ten modified forms of H2A (Figure 3B). Acetylated serine one (S1ac) had the most modified product fragments ions detected in the MS2 and the highest relative intensity. Interestingly, S1ac appeared to trend lower in both number and intensity as Teff migrated to the infection site (Figure 3B). The same held true for H2A S1 phosphorylation (H2AS1p), which was previously associated with repression in embryonic development [57,58] and chromatin relaxation to enhance transcription ribosome biogenesis and control of cell growth [59,60]. H2AS1p was accompanied by nearby R3me2 and K5ac, which were higher in the spleen than in the lung, and not detected in BAL (Figure 3B). It is notable that R3me2 occurred in combination with H2A S1p, not S1ac, as acetylation at neighboring lysine residues is known to block methylation of arginine eight on H3. It is also remarkable that proteoforms with single R3me2 modifications were highly abundant in BAL, given proteoforms with R3me2 and S1p were only detected in Teff from the spleen. H2AK5ac was highly abundantin the lung, both as a single modification and accompanying S1ac (Figure 3B). This combination is intriguing because S1ac blocks phosphorylation at this site, which is a repressive mark, and K5ac is associated with transcriptional activation [61,62,63,64]. These acetylation events were accompanied by T120p, which is associated with increased gene expression and genomic stability in replicating cells [65,66]. By contrast, H2B had fewer PTMs, and the majority of species detected were unmodified, irrespective of Teff location (Figure 3C). H2B phosphorylation at serine 14 (S14p) is considered an epigenetic marker of apoptotic cells that works in opposition to the adjacent K15ac present in non-dying cells [67]. We detected some S14p in the spleen and lung (Figure 3E). Lysine 23 acetylation was the only modification we detected on H2B in Teff from BAL, while lysine 108 acetylation was detected only in Teff from the lungs. Lysine 108 acetylation has been widely reported in other tissues and systems, but its function remains unknown [68,69,70,71].

**Figure 4:**
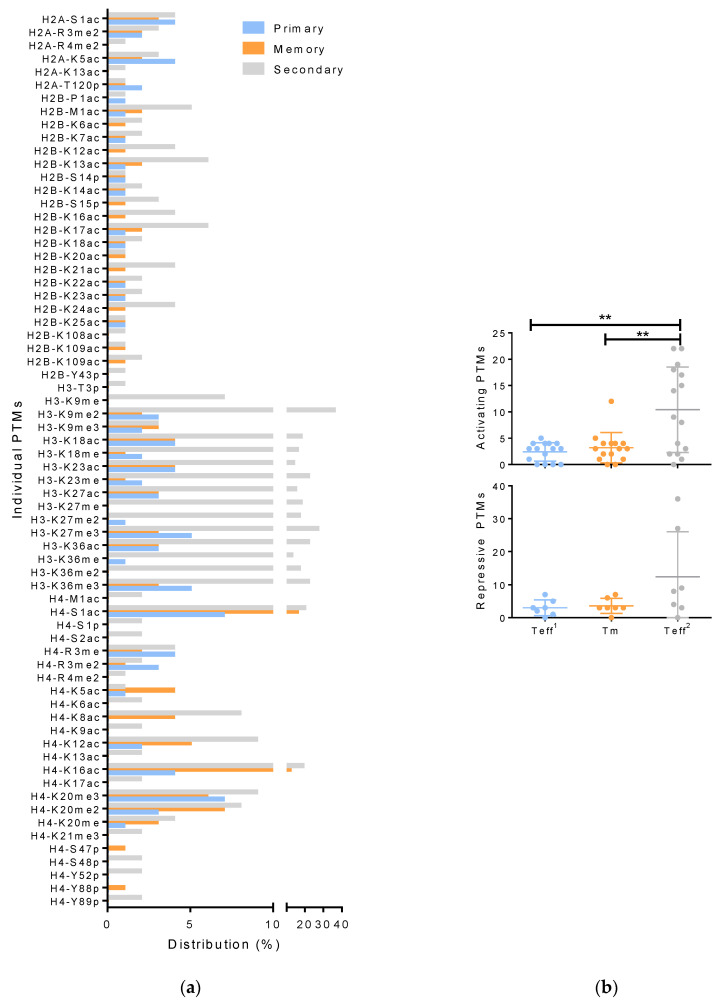
HiPTMapping of primary, memory, and secondary CD8 T cells. (**a**,**b**) Histones were purified from T cells and subjected to RPLC–MS/MS using Orbitrap Velos with alternative CID/ETD. Raw datasets were searched against the mouse top-down database. PTMs including acetylation; mono-, di-, and trimethylation; and phosphorylation were detected. The occurrence of each modification per proteoform was combined to yield the sum of each specific PTMs per core histone. (**a**) ANOVA (** *p* < 0.0001) was performed followed by Tukey’s multiple comparisons test: Teff^1^, vs. Tm (*p* = 0.17), Tm vs. Teff^2^ (*p* < 0.0001), and Teff vs. Teff^2^(*p* < 0.0001); (**b**) PTMs were separated into known activation marks (H2A-S1p, H2A-K5ac, H3-K36me2, H3-K9ac, H3-K27ac, H2B-K12ac, H3-K18ac, H3-K23ac, H3-K36ac, H3-K36me3, H4-K5ac, H4-K8ac, H4-K12ac, H4-K8ac, H4-R3me2, and H4-K16ac) and repressive marks (H3-R8me2, H3-K9me2, H3-K9me3, H3-K27me3, H4-K20me2, H4-K20me3, H4-K20me). Statistical comparison was with ANOVA (*p* = 0.007) followed by Tukey’s multiple comparisons test (*p* < 0.005).

In contrast, H3 was heavily modified, many were bivalent, and these proteoforms were most abundant in Teff from the spleen and BAL (Figure 3D). Of all the core histones, H3 exhibited a stark pattern of PTMs changes based on Teff location (Figure 3D). Again, due to the higher complexity of H3, the normalized intensity of these proteoforms was relatively low. However, we identified many proteoforms that corresponded to numerous fragment ions. We did not detect H3K4me3, but this site was monomethylated and was detected only in Teff from the BAL (Figure 3D). Active gene loci are characterized by a combination of all three methylated forms of H3K4 and H3K4me, and H3K4me2 are found at both transcriptionally active promoters and distal regulatory elements and are considered activating signals [72,73,74,75]. Both H3K4me3 and H3K27me3 play an essential role in the polarization and activation of immune cells, including CD8 T cells [76,77,78,79,80,81]. We found repressive K27me3 on nine different H3 proteoforms combined with many other PTMs (Figure 3D). In addition, repressive K9me3 was present in 12 out of the 23 proteoforms we confidently identified. Interestingly, we identified several H3 proteoforms with K9 methylations and K27 di- and trimethylations that were exclusively identified in Teff from BAL, especially when PTMs were absent in the intervening region (Figure 3D). This combination is also known for controlling the lineage commitment of both CD4 and CD8 T cells [82,83]. H3K18ac is associated with increased transcription in T cell activation [81,84]. H3K18ac was almost exclusively detected in Teff from the spleen with hyper-acetylation of neighboring lysines (Figure 4). Thus, while H3 wass of lower abundance, we found several modifications alone or in combination that are associated with T cell activation and lineage commitment. Given this included a large number of PTMs surrounding the well-studied H3K4me3 and H3K27me3 regulatory marks and the apparent enrichment of these species in different locations, exploring how these combinatorial patterns impact gene expression may be worthwhile.

Similar to H3, the tail region of H4 had multiple PTMs combinations, and most were bivalent (Figure 3E). The majority of these proteoforms were detected at a similar frequency and relative intensity in Teff from the BAL, lung, and spleen. The only exception was S1p, accompanied by K20me3, which was found exclusively in Teff from the BAL, was highly abundant (9%) and had several matched fragments (Figure 3E). Like acetylation, phosphorylation of histone tails alters their charge and changes electrostatic interactions to relax chromatin. H4S1p has been implicated in the regulation of gene expression and control of cell phenotypes as well as decreasing histone acetyltransferase activity [85,86]. We also detected several H4 proteoforms with S1 acetylation. Both S1p and S1ac were associated with T cells from the BAL. We also identified several H4 proteoforms with R3me2; R3me2 is associate with the loss of S1p. Interestingly almost all H4 proteoforms had either S1 modification or R3me2, and these modifications were never present on the same proteoform (Figure 3E). This may indicate, in addition to phosphorylation, R3me2 can also block acetylation at serine one. However, R3me2 was detected in combination with several other modifications, including K12ac and K16ac and K20me2 and K20me3 (Figure 3E). These H4 lysine modifications alter binding affinity and may play a role in enhancing or inhibiting regional regulatory complexes [87]. We also identified two other modifications, K5ac and K12ac, that are associated with transcriptional activation [61,62,63]. Taken together, these data indicate a previously unappreciated role for histone modification dynamics in Teff during migration to infection sites.

### 3.3. Histone Modifications in Memory and Effector T Cells Following Influenza Infection

There are fundamental differences in the response of naïve versus educated T cells to infection. In response to primary infection, T cell expansion peaks around day 9 or 10 days. This is followed by contraction, leaving 5–10% of antigen-specific CD8 T cells to form the remaining pool of memory cells. In a subsequent secondary infection, these cells expand more robustly and mediate viral clearance faster. We compared the histones from Teff on day 9 of the primary response to X31 (Teff^1^) to early central Tm on day 30 and to activated T cells collected 7 days following a heterologous challenge (Teff^2^). All mice were intranasally infected with influenza strain X31. However, for the secondary response, mice were primed with PR8 influenza strain at least 30 days prior to infection with X31 (Figure 1). We used the heterologous PR8/X31 prime challenge model because in mice, a homologous challenge results in sterilizing immunity without appreciable T cell expansion. The X31 and PR8 strains express different surface HA and NA proteins, so this prime challenge model avoids cross-reactive neutralizing antibodies. There is some naïve response to the heterologous HA and NA, but these Teff^1^ lag behind Teff^2^ [88]. This is due to the conservation of the five dominant influenza epitopes in the internal genes of X31 and PR8. Thus, the Tm are already primed with PR8, and their expansion dominates the secondary response. Supplementary File S1 tabs B, C, and D contains the complete list of observed species, their masses, and *p*-values for Teff^1^, Tm, and Teff^2^, respectively. We compared the occurrence of individual PTMs per condition (Figure 4). Histones from Teff^2^ had significantly more modifications than histones from Teff^1^ or Tm (Figure 4a). Interestingly, the transcriptional activation mark H2AK5ac [62,63] was similar for all subsets (Figure 4a and Table 2). Dimethylation of arginine 4 and acetylation of lysine 13 are known to target this area for biotinylation, which is involved in cell proliferation [89,90]. H2A R4me2 and K13ac were detected only in Teff^2^ (Figure 4a and Table 2). Apart from R4me2 and K13sc, PTMs on H2A appear in all three T cell subsets (Figure 4a).

When we compared modifications to H2B, we found Teff^2^ showed a high distribution of almost all proteoforms identified (Table 3 and Figure 4a). We identified P1ac on the H2B proteoforms in the Teff^1^. When accompanied by a string of acetylations in this region, P1ac was detected in the Teff^2^ (Table 3). In contrast, acetylations at residues 16, 17, 18, 22, and 24 were detected in all three T cell subsets. Acetylations at lysine 7, 12, 13, and 14 were largely associated with Teff^2,^ and Y42p on H2B were detected only in Teff^2^ (Figure 4a). Phosphorylation of serine 14 (S14p) on H2B is correlated with somatic hypermutation in vivo and class switch recombination [91]. We found H2BS14p was consistent across all three subsets (Figure 4a and Table 3). In contrast, K20ac was detected in Tm and Teff^2^ subsets (Table 3). This modification was previously detected on H2B [92] and was recently implicated as the site-specific homing and docking point for macroH2A1, which is required for the transcriptional activation of a myriad of cytokines, chemokines, metalloproteases [93]. Acetylation at lysine 108 or 109 in the tail region was also restricted to Tm and Teff^2^ (Figure 4a).

We identified very few H3 proteoforms in Tm (Table 4 and File S1C). However, all H3 proteoforms identified in Tm were bivalent. We found methylation of lysine 9 and 27 on H3, and these repressive modifications were colocalized in three proteoforms from Teff^1^ versus twelve in Teff^2^. In general, these repressors were found in combination with activation marks. H3K27ac and H3K36ac are enhancer signals [94] that were also highly abundant in Teff^2^ (Figure 4a). However, lysine 36 can be methylated, which is a repressive signal [95]. H3K36 methylation was also highly abundant in Teff^2^ (Figure 4a). The most striking differences in individual PTMs on H3 were observed in regions 9 to 37, where these PTMs occurred in a variety of combinations (Figure 4a and Table 4). These unique proteoforms included several known enhancer and repressor signals (K4ac, K9me, K18ac, K23ac, K36ac, and K37me, [61,63,96,97,98,99,100,101] and R8me, K9me, and K27me [102,103,104,105,106], respectively). In some cases, H3 proteoforms differed by modifications with opposing functions on a single amino acid. For example, K9, as well as K36, can be acetylated or methylated to induce activation or repression of gene expression at nearby loci [107,108]. We also found evidence of bivalent “super-enhancers” with multiple activation signals on neighboring residues as well as a repressor signal in the flanking regions along with traditional bivalent marks. The abundance of H3 species with activation and repression marks indicates these histones may be poised to respond to external condition changes in these states. Indeed, all of the proteoforms from Tm were bivalent. In contrast, all of the H3 proteoforms with exclusively enhancer or repressor marks were detected in Teff, with a higher number in Teff^2^ (Table 4). Furthermore, the diversity and abundance of PTMs on H3 proteoforms in the secondary response support the notion that PTMs accumulate in terminally differentiated T cells.

H4 had fewer unique combinations of modifications than H3, but most H4 proteoforms were detected in Tm (Table 5). Serine 1 was acetylated in all T cell subsets but occurred in 80% of H4 proteoforms identified in Tm and in 43% and 32% of PTMs combinations from Teff^1^ and Teff^2^, respectively. S1 was also phosphorylated, but this modification was unique to Teff^2^ (Figure 4a and Table 5). This phosphorylation event is permissive and associated with proliferation [57]. S1p was accompanied by arginine 3 methylation (R3me), which is also an enhancer of gene expression [99,109,110]. R3me was present in both primary and secondary responses on multiple H4 proteoforms in combination with other activators and the repressive mark K20me [96,111,112,113]. H4 K5ac and K8ac are activation marks [61,62,63,114,115] that were associated with Teff in both the primary and secondary responses (Table 5). Nearby, lysine 12 and 16 were also acetylated, and these are transcriptional activation signals [61,62,63,114,116]. H4 K12ac and K12ac were present in all subsets but were much lower in Teff^1^ (Figure 4a). Acetylation of lysine 9, another activation mark [53,54], was only detected in Teff^2^ (Figure 4a). Similar to H2, these activating acetylations were grouped closely together. Interestingly, K16ac was often accompanied by K20 di and trimethylation. These repressive marks were on approximately 50% of H4 proteoforms from Teff^2^ versus 75% and 88% of the H4 PTMs combinations from Teff^1^ and Tm subsets, respectively. Similar to H3, we found an abundance of H4 bivalent proteoforms that combined activating marks with the repressive K20me2 and K20me3. This was the only known repressor we identified on H4. However, it should be noted that mono-methylation of K20 can enhance or repress gene expression [96,111,112,113]. Thus, we found a myriad of histone proteoforms with unique combinations; some were shared among T cell subsets while others were unique or enriched in specific subsets. When we compared only the canonical enhancer and repressor PTMs, we found Teff^2^ had significantly more PTMs associated with activation than Teff^1^ or Tm (Figure 4b).

## 4. Discussion

Histone modifications in the respiratory tract have been associated with respiratory inflammation and infection, including SARS-CoV [117,118]. Influenza induces modifications that alter the expression of some pro-inflammatory cytokines and interleukin genes, but this is largely associated with DNA methylation during viral replication [119,120,121,122,123,124,125,126]. Thus, the epigenetic changes we observed in T cells are not likely to induce by the influenza virus itself. We found histone PTMs profiles differed in Teff isolated from the spleen, lung, and BAL following influenza infection (Figure 3). Others have demonstrated that location can impact epigenetic programming in innate immune cells and CD8 T cells [127,128]. We posit this is due to their distinct microenvironments and may enhance functional differences in the T cell subsets at discreet locations. The interstitial T cells of the lung and airway T cells in the BAL are functionally different (e.g., more cytolytic versus more rapid cytokine response, respectively), and the quantity of BAL Teff correlates with protection during influenza infection. Recently, using the same PR8-X31 prime challenge model, Hayward et al. assessed the chromatin accessibility of influenza-specific T cells from the lung and BAL [128]. They found coordinated changes in chromatin accessibility and gene expression that suggested their epigenetic architecture was influenced by their environment and concluded amino acid starvation was the underlying mechanism. However, we interpret changes in amino acid starvation pathways to be downstream of the metabolic response to changes in substrate concentration in the microenvironment. Metabolism is sensitive to nutrient concentrations and exerts direct and indirect control of epigenetic programming [129,130,131,132]. T cell subsets have distinct metabolic phenotypes that may render them more sensitive to the availability of nutrients in their microenvironment, and this is likely to contribute to location specific epigenetic programming.

Previous studies profiled PTMs at certain effector gene loci and determined these marks were associated with genes that are critical to subset specific T cell functions, including human naive and memory CD8 T cells. These studies found associations between gene expression and the amounts of H3K4me3 (positive correlation) and H3K27me3 (negative correlation) across the gene body [81,133,134]. Araki and co-workers defined 4 associations between the ratios of H3K27me3 and H3K4me3 and gene expression: repressed, active, poised, and bivalent (i.e., high and low with low expression, low and high with high expression, low and high with low expression, and high and high with constant low expression in Tn that increases when Tm are activated) [81]. A similar approach demonstrated, after in vivo differentiation into influenza-specific Teff, H3K27me3 decreased, and H3K4me3 increased at immune-related effector gene promoters [3]. We used an unbiased approach and did not identify H3K4me3 or mine the data to identify or quantify this species. However, we found a reduction in H3K27me2 and H3K27me3 repressive marks that were associated with T cell activation (Figure 2b and Table S1). We identified more unique combinations of PTMs from naïve than activated T cells isolated from the same samples, as well as significantly more individual PTMs and known markers of transcriptional activation (Figure 2b inset). Moreover, after a secondary recall response to the heterologous influenza challenge, activation of Tm significantly increased the number of PTMs known to mediate transcriptional activation compared to naïve, Teff^1^, or Tm. This was largely due to several internal acetylations of lysine 9, 18, 23, 27 and 36 on H3 associated with transcriptional activation [135]. This was complemented by di- and trimethylation at lysine 36, a strong enhancer of transcriptional activation in T cells. Indeed, increased chromatin accessibility via hyperacetylation of this region is a potentially important mechanism for rapid re-expression of effector genes that facilitates the unique capacity for rapid recall of effector CD8 T cells [24]. Araki and Russ et al. established that H3K4me3 and H3K27me3 are reliably associated with genes that regulate T cell differentiation, and function via these marks positively and negatively correlating with the expression patterns of many critical genes and established methylation dynamics at bivalent regions regulate Tm by making these gene loci poised for rapid expression [3,81]. Our findings add to this knowledge by defining new PTMs combinations and deciphering the histone code by mapping these epigenetic hot spots in distinct CD8 T cell subsets over the course of in vivo influenza infection. We identified many new bivalent combinations in T cell subsets and found significant differences in the distribution of individual PTMs with activation and across T cell subsets and significantly more enhancer PTMs in Teff^2^.

**Figure 5:**
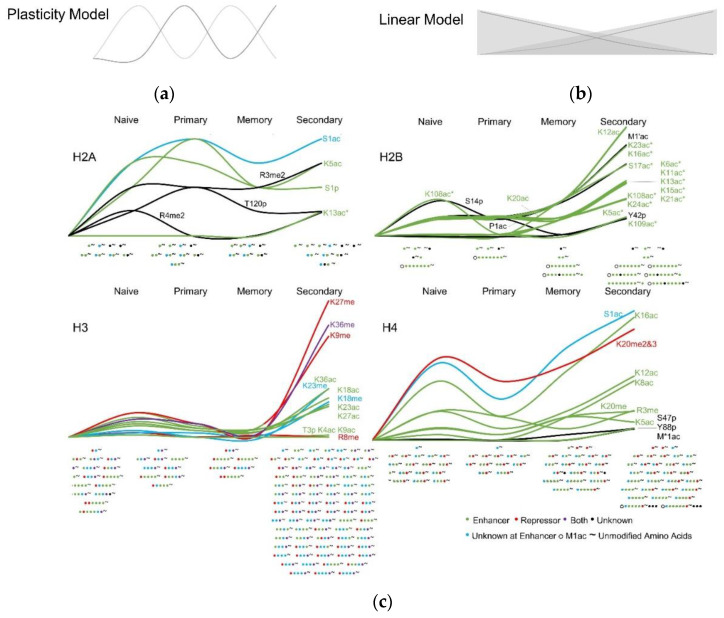
Patterns of histone PTMs over time with CD8 T cell differentiation after influenza infection. T cells have unique histone PTMs that are thought to form based on two models of T cell differentiation. (**a**) In the plasticity model, combinations of PTMs oscillate in on and off patterns as T cells progress through differentiation. In this case, the naïve T cells are activated and differentiate into effector T cells in the primary infection and are either terminally differentiated and contract or form Tm that are activated in the secondary infection and follow the same cycle. In this model, the PTMs cycle in on and off and on patterns regulating similar patterns of transcription. (**b**) In the linear differentiation model, T cell progression from naïve to primary or memory is mediated by the persistence and magnitude of antigenic stimulation. Effector cells that are not fully differentiated when the activation signal wains remain as memory cells in a transitory state of mixed on or off signals that are driven in one direction or the other in the secondary infection. In this model, there is a progressive gain or loss of enhancing and repressing PTMs. (**c**) The presence of individual PTMs in the identified proteoforms was tallied for each T cell subset and graphed. Below the x-axis, each unique combination of PTMs is represented per naïve, primary, memory, and secondary states. PTMs are presented as circles, and the tilde symbol (~) indicates unmodified stretches of amino acids. Circles to the left of the tilde are in the amino-terminal histone tails, and on the right are at the carboxy terminus of the histone.

Based on the correlations of H3K4me3 and H3K27me3 with gene expression profiles in T cell subsets detailed above, many have integrated these epigenetic patterns into the two current models of T cell differentiation [136,137,138]. The circular T cell differentiation model suggests plasticity in T cells such that memory precursors, formed early after activation, gain effector function while maintaining the ability to form a pool of memory subsets. In this cycle, Tn differentiate into effector T cells, and a few develop into Tm that are activated in subsequent infections to repeat this process. It is postulated that the cycles of differentiation, dedifferentiation, and redifferentiation would result in oscillations in gene expression as well as epigenetic marks [136,137,138] (Figure 5a). In the linear differentiation model, naïve cells are fully activated into effector T cells that contract while memory subsets form from naïve cells that are activated at the end of the infection and do not receive the necessary signaling to form Teff. Thus, it is postulated that the transcriptional and epigenetic programs of these transitional Tm will overlap with Tn and Teff and accumulated on or off signals resulting in a progressive increase or decrease in gene expression and related epigenetic marks [138] (Figure 5b). Presumably, these accumulated on or off signals would then be amplified in the secondary response. There is substantial evidence to support both differentiation models, and they remain a contested topic in T cell biology. To determine if individual histone PTMs fit these models, we used a qualitative approach. We determined the presence of each PTM and considered its accumulation based on the number of times that modification was identified alone or in unique PTM combinations for each T cell subset (i.e., unique proteoforms with the same PTMs combinations were combined, and we did not account for the number of times a proteoform was identified, the number of corresponding fragments, or their intensities). Using this high level on/off and up/down approach, we found evidence that some PTMs are constant while others oscillate in frequency or accumulate as T cells differentiate (Figure 5c).

The circular or oscillation model fit for repressive modifications H3 R8me and H4 K20me, which had on–off–on–off and high-low-high-low prevalence, respectively. Similarly, H4 S1ac and the activators H4 K12ac and K16ac oscillated from high to low to high in Tn, Teff^1^ and Tm, respectively, and remained high in Teff^2^ (Figure 5c). The methylations of H4 R3 and H3 K27 and K36 were detected less frequently in Tm, resulting in Tn, Teff^1^, Tm, and Teff^2^ following a high-high-low-highest pattern (Figure 5c). Following the linear accumulation model, some PTMs were acquired with memory and maintained or increased prevalence in the secondary response (H2B K5ac, K11ac, K15ac, and K20ac and H4 S47p and Y88p) (Figure 5c). These were often associated with neighboring modifications that were present at low levels in Tn and Teff^1^ but increased at the memory stage (e.g., H2B K12ac, K16ac, and K23ac and H4 K5ac and K20me). Acetylation of lysine 13 and 109 was detected on H2A and H2B only in the Teff^2,^ as was phosphorylation of H2B Y42 and H4 S1 and Y51 (Figure 5c). Acetylation of lysine 6, 13, 17, and 24 on H2B was only detected on one proteoform until the secondary response when it was detected in several PTMs combinations. Methylation of K18 on H3 followed a similar trend. Modifications that did not fit these models may have been influenced by the heterogeneity of the Tn and Teff^2^ populations. For example, a few modifications were detected exclusively in the Tn and Teff^2^ (i.e., H2A R4me2 and H3T3p, and K23me). All the acetylations to the mid-region of the amino-terminal tail of H3 (i.e., K18-36) occurred at a higher frequency in Tn and Teff^2^. Methylation of K9 on H3, a repressor, also followed this pattern (Figure 5c). Interestingly, the opposing activating acetylation of this lysine and nearby lysine 4 was only detected in naïve T cells, and these were the only PTMs that were unique to this subset.

This study is limited in that it does not relate these modifications to gene expression. Instead, it provides the identity and abundance information for specific histone modifications and their combination per proteoform in T cell subsets isolated from mice over the course of influenza infection. The high-level analysis summarized in Figure 5 supports the current models that attempt to integrate epigenetics into T cell differentiation paradigms. Previously, these models integrated naïve T cells with T effector cells in the primary response and Tm subsets. Here we provide additional clarity on the epigenetic programming of Teff in the secondary response. However, these were bulk T cell populations within each subset as we were unable to purify enough histones from antigen-specific T cells to compare across groups (DNS). In lieu of tetramer staining, we employed a gating strategy to increased influenza-specific populations in the BAL, lung and spleen [10]. In the primary response, influenza-specific CD8 T cells are the dominant population. Due to the five dominant epitopes shared between X31 and PR8, it is even higher in the secondary response (between 70 and 90%) [10,88,139]. T cell heterogeneity may contribute to the higher accumulation of a unique combination of H3 and H4 PTMs in Teff^2^ (Figure 5c). However, T cell heterogeneity in the naïve population is much higher than either Teff^1^ or Teff^2^, and the histone PTMs diversity from Tn is lower than Teff^2^. Thus, it is unlikely heterogeneity of Teff^2^ accounts for the diversity in the histone proteoforms. It is feasible these PTMs combinations contribute to the enforcement of terminal differentiation.

We concede these data may not be generalizable to discreet subpopulations within each compartment. Nonetheless, the permissive and repressive marks appear to be maintained in the generation of daughter cells from the memory pool and increased in the secondary response. Perhaps the most notable of these were the group of H2B and H4 PTMs mentioned above that were either acquired or increased in Tm and sustained Teff^2^. Consistent with previous reports [140], we saw a decommissioning of some naïve enhancer PTMs as well as the generation of new enhancers in Teff^1^ and Teff^2^ (Figure 5c). Like previous reports, the H3 modifications we identified in Tm were bivalent. We expected to see a loss of repressor signals with a gain of enhancers in Teff^2^. Instead, we saw an increase in some repressors and a significant increase in enhancer marks in Teff^2^ (Figure 5). In the case of H3K9 methylation, this makes good sense as it is associated with the short life span of CD8 effector cells [141]. The bulk of what is known relating gene expression patterns to histone modification in T cell subsets centers around H3K4me3 and H3K27me3. We did not detect H3K4me3, yet our data suggest other combinations and modifications may be worth exploring. Indeed, in the studies mentioned above, gene expression did not always correlate with H3K4me3 and H3K27me3 patterns and appear to be best associated in terms of H3K4me3 at effector loci in Teff^1^ [17,18,81,142,143]. This suggests other mechanisms may be influential in maintaining epigenetic programming throughout the stages of T cell differentiation and recent research in this area indicates even minor changes in highly conserved regions alter chromatin structure and gene regulation [144,145]. One possibility is that unique PTM combinations, especially those including understudied modifications that block or induce regional modifications, act as tuning mechanisms or overriding signals that functionally contribute to the role of the histone code in controlling T cell fate.

Due to their high heterogeneity, histones are inherently challenging to study. In addition, interpreting combinations of histone PTMs is difficult because of the lack of knowledge regarding their biological function [146,147,148]. However, our identification of these differentially marked histones in unique combinations from distinct locations and T cell subsets raises several important questions. First, are microenvironmental conditions influencing T cell epigenetics and is this functionally relevant? Second, are the combinations of histone PTMs we identified controlling gene expression in the T cell response, or do they play other currently undefined roles such as regulating regional modifications? If the former is true, PTMs may act to fine-tune canonical modifications or function independently. Third, are the modifications acquired with memory and sustained in the secondary response mediating memory cell survival or necessary for homeostatic proliferation upon recall? HiPTMap of activated versus naïve T cells subsets and effector subsets across locations acquired from the same groups of mice as well as the comparison of T cells over time following influenza infection has provided the blueprint to answer some of these questions and will likely contribute to our understanding of epigenetic regulation of T cell biology.

## 5. Conclusions

The HiPTMap strategy used here enhances proteoform identification, providing a comprehensive analysis. Our results show the high potential of online nanoflow 1D and 2DLC separations for top-down analysis of histones and HiPTMap strategy. In addition, this platform extended the dynamic range of MS measurements, increasing the number of confidently identified histone proteoforms compared to 1D LC-MS approach. With this strategy, we identified hundreds of unique histone-modified species isolated from mice following influenza infection. As a result, we now know that several new bivalent combinations are present in effector T cells; the histone map changes as T cells relocate during influenza infection; and the secondary recall response of CD8 T cells induces a significant increase in histone PTMs associated with transcriptional activation. These histone modifications appear to oscillate or accumulate over the course of T cell differentiation in a PTMs specific manor. Recent advancements in top-down technology will facilitate more confident and comprehensive histone proteoform characterization and enable us to approach (near) single-cell sized samples to better address cell heterogeneity challenge. Furthermore, computational approaches are rapidly advancing [48,149,150], and we anticipate even more detailed data-mining to be achieved from HiPTMap with the analysis that will result in the discovery of interdependent relationships between unique modified forms identified here and reported elsewhere to decipher the histone code.

## Figures and Tables

**Table 1:** Number of unique modified sequences identified from naïve and activated CD8 T cells.

		H1F	H2A	H2B	H3	H4	Total
Naive	Identified		30	18	18	25	91
	Fragments		832	443	291	598	2164
	Unique		9	6	16	19	50
Active	Identified	1	39	12	10	13	75
	Fragments	21	635	356	139	279	1430
	Unique	1	10	5	9	12	36

The number of proteoforms identified, the sum of their corresponding fragments ions detected, and the total number of unique modification combinations.

**Table 2:** H2A modifications from Teff^1^, Tm, and Teff^2^ after influenza infection.

H2A	Teff^1^		Tm		Teff^2^	
	Intact ID	Frag (#)	Intact ID	Frag (#)	Intact ID	Frag (#)
S1ac	15	377	21	304	26	749
S1p	2	31	2	26	4	75
R3me2	1	10	1	1	3	13
R4me2					1	20
K5ac	6	6	6	6	5	5
S1acK5ac	4	53	2	59	5	95
S1acR3me2K5ac					2	21
S1acK5acT120p	1	11				
S1acT120p	4	54	1	1	5	98
S1pK5ac	2	18				
S1pR3me2	1	10	1	9	3	74
K13ac					1	12
None	2	65	5	155	8	158

Intact ID: the number of intact proteoforms identified containing listed modification(s). Frag #: the number of fragment ions detected for the proteoforms.

**Table 3:** H2B modifications from Teff^1^, T_M_, and Teff^2^ after influenza infection.

H2B	Teff^1^		T_M_		Teff^2^	
Modifications	Intact ID	Frag (#)	Intact ID	Frag (#)	Intact ID	Frag (#)
P1ac	1	17				
P1acK6acK12acK13acK16acK17acK21acK24acK109ac					1	15
M’acK5acK11acK12acS14pK15acK16acK20acK23acY42p					2	59
M1′acK6acK12acK13acK16acK17acK21ac					1	23
M’acK6acK12acK13acK16acK17acK21acK24ac	1	18	1	24	2	56
M’acK11acK12acS14pK15acK16acK20acK23ac					1	28
M’acK11acK12acS14pK15acK16acK20acK23acK108ac			1	15	1	16
S14p	1	24	2	35	5	135
K20acK23ac			3	31	8	310
K23ac	1	21			7	259
K108ac					2	83
None	8	267	10	286	22	804

Intact ID: the number of intact proteoforms identified containing listed modification(s). Frag #: the number of fragment ions detected from the proteoforms. M’ N-terminal initiator methionine.

**Table 4:** H3 modifications from Teff^1^, T_M_, and Teff^2^ after influenza infection.

H3	Teff^1^		T_M_		Teff^2^	
Modifications	Intact ID	Frag (#)	Intact ID	Frag (#)	Intact ID	Frag (#)
T3pK9me3K27me3K36me					1	11
R8me2K9meK18acK23acK27acK36ac			1	10		
K9me2K18me					1	29
K9meK18meK23meK27me					1	32
K9meK18meK23meK27meK36me			1	10	2	58
K9me2K18meK27me					1	27
K9meK18meK27me2K36me					4	124
K9me2K18meK36me3					1	22
K9me2K18meK23acK27acK36ac	2	29			1	28
K9meK18meK27meK36ac					2	61
K9meK18acK23acK27acK36ac	1	8	2	26		
K9me2K18acK23acK27me3K36me3	1	13	1	9	1	25
K9me2K18acK36ac					1	24
K9me2K23meK27me					2	58
K9meK23meK27meK36me	1	13			4	98
K9me2K23meK36me3					1	26
K9me2K23meK27meK36ac					1	31
K9me2K23acK27acK36ac					1	24
K9me2K23acK27me3K36me3					2	47
K9me3K23acK27acK36me3					1	24
K9me2K27me					6	168
K9meK27meK36me	1	18	1	7	17	501
K9me2K27ac					2	53
K9me2K27acK36me					2	47
K9me2K27acK36ac					1	29
K9me2K27me2K36ac					3	76
K9me2K36ac					3	77
K9me2K36me					4	110
K18meK23meK27me3K36me3	1	13			1	26
K18acK23meK27me3K36me					2	49
K18acK23acK27acK36ac	1	14			1	23
K18acK23meK27acK36ac					2	56
K18acK23acK27meK36ac					1	26
K18acK23acK27meK36me					4	109
K18acK23meK27me2K36ac					1	22
K18acK23meK27me3					1	32
K18meK27me3K36me3					1	16
K18acK27acK36ac					2	52
K18acK27acK36me					4	100
K18acK27meK36me	1	20			2	61
K18acK27meK36ac					2	48
K23meK27me3					3	72
K23meK27me3K36me					7	185
K23meK27meK36ac					2	60
K23acK27me					1	31
K23acK27me3K36ac					1	22
K23acK27meK36me					4	107
K27acK36ac					5	137
K27acK36me					4	119
K27me2					1	30
K27meK36me	1	11			10	268
K27meK36ac					3	89

Intact ID: the number of intact proteoforms identified containing listed modification(s). Frag #: the number of fragment ions detected for the proteoforms. Underline indicates methylations were combined.

**Table 5:** H4 modifications from Teff^1^, T_M_, and Teff^2^ after influenza infection.

H4	Teff^1^		T_M_		Teff^2^	
Modifications	Intact ID	Frag (#)	Intact ID	Frag (#)	Intact ID	Frag (#)
S1ac	1	17			1	15
S1pR3me					1	6
S1pR3meK20me3					2	23
S1acR3meK20me2			1	14		
S1acK5acK8acK12acK16ac					2	22
S1acK5acK8acK12acK16ac			1	19		
S1acK5acK8acK12acK16acK20me2			1	13		
S1acK5acK8acK16acK20me			1	23		
S1acK5acK12acK16acK20me			1	27		
S1acK8acK12acK16ac					2	43
S1acK8acK12acK16acK20me			1	56	3	48
S1acK8acK16acK20me					5	135
S1acK12acK16ac					3	69
S1acK12acK16acK20me	1	13	2	78	7	202
S1acK16ac			1	12	4	79
S1acK16acK20me	2	29	5	191	10	301
S1acK16acK20me3S47p			1	9		
S1acK20me	4	115	6	241	9	266
S1acK20me3Y88p			1	19		
R3me2K12acK20me3	1	25				
R3me2K16acK20me	1	21			1	10
R3meK20me	2	47	1	23	2	25
K5acK20me3	1	12				
K16ac			1	13		
K16acK20me					2	31
K20me			2	97	2	34
M1acS2acK6acK9acK13acK17acK21me3-S48pY52pY89p					1	6
M1acS2acR4me2K6acK9acK13acK17ac- K21me3S48pY52pY89p					1	6
None					2	24

Intact ID: the number of intact proteoforms identified containing listed modification(s). Frag #: the number of fragment ions detected for the proteoforms. Underline indicates mono, di, and trimethylation were combined.

## Supplementary Materials

The following are available online at https://www.mdpi.com/1999-4915/12/12/1409/s1, Table S1: Modified core histone proteoforms identified in Tn and activated CD8 T cells. Table S2: Relative abundance of histone modifications from naïve and activated CD8 T-cells. File S1: Summary LC-MS data of T cell subsets after primary and secondary influenza infection. File S2: Summary LC-MS data of histones from activated CD8 T cells from the spleen, BAL, and lung after influenza infection.
