# Supplementary material for: Mapping Influenza-Induced Posttranslational Modifications on Histones from CD8+ T Cells
Source: Viruses. 2020 Dec 8;12(12):1409. doi: 10.3390/v12121409 (PMC7762524; doi:10.3390/v12121409)
Supplement: Supplementary file 1 [file viruses-12-01409-s001.zip › supplementary/201112_Final Supplementary tables.pdf]

Supplementary Table 1. Modified core histone species identified in naive and activated CD8+ T cells.

Core Histone	Modifications Identified	Naïve Unique Sequences	Naïve Detected Fragments	Active Unique Sequences	Active Detected Fragments
**H1F**	S1ac			1	21
**H2A**	K5ac			6	6
	none	4	102	3	65
	R3me2	3	32	1	10
	R4me2	1	24		
	S1ac	12	491	15	377
	S1acK5ac	1	29	4	53
	S1acK5acT120p			1	11
	S1acT120p	3	60	4	54
	S1p	3	38	2	31
	S1pK5ac	2	48	2	18
	S1pR3me2	1	8	1	10
**H2B**	K108ac	1	25		
	K23ac	1	34	1	21
	M1acK7acK13acK14acK17acK18acK22acK25ac	1	23	1	18
	none	13	343	8	276
	P1ac	1	12	1	17
	S14p			1	24
	S14pK108ac	1	6		
**H3**	T3pK23acK27me2K36me2	1	12		
	K4acR8me2K9acK18acK23acK27me3K36me3	1	11		
	R8me2K9meK18acK23acK27acK36ac	2	29		
	K9meK18meK27me2K36ac	1	22		
	K9meK18acK23acK27acK36ac	3	46	1	8
	K9me2K18acK23acK27me3K36me3	1	19	1	13
	K9me2K18meK23acK27acK36ac	1	14	1	29
	K9me2K23acK27me3K36me3	1	19		
	K9me2K27me2	1	26		
	K9meK27me3K36me3	1	11	1	18
	K9me3K23meK27me3K36me3	1	12	1	13
	K9me3K27meK36me2	1	21		
	K18acK23acK27acK36ac			1	14
	K18acK23meK27me3	1	15		
	K18acK23meK27me3K36me	1	15		
	K18meK23meK27me3K36me3			1	13
	K18acK27me2K36me			1	20
	K23acK27acK36ac	1	19		
	K27me3K36me3			1	11
**H4**	S1ac	1	13	1	17
	S1acK12acK16ac	1	13		
	S1acK12acK16acK20me	2	29	1	13
	S1acK16ac	1	17		
	S1acK16acK20me	3	169	2	29
	S1acK20me	6	231	4	115
	S1acR3meK20me	2	30		
	R3meK20me3			1	28
	R3me2K12acK20me3			1	25
	R3me2K16acK20me	1	9	1	21
	R3me2K20me3	1	32	1	19
	R3me2K5acK8acK20me2	1	8		
	K5acK20me	1	21	1	12
	K12acK16acK20me	2	26		

Total number of confidently matched sequences and corresponding fragments detected per proteoform. Underline denotes di and tri methylation combined

Table 2. Relative abundance of histone modifications from naïve and activated CD8+ T-cells.

Core Histone	Isoform	Naïve	Active
H2A	Total (%)	25.0	55.4
	Unmodified	5.2	2.5
	S1ac	6.5	23.2
	S1acK5ac	0.2	3.4
	S1acK5acT120p		0.8
	S1acT120p	2.9	3.3
	S1p	6.1	10.9
	S1pK5ac	0.6	1.0
	S1pR3me2	0.4	9.4
	R3me2	3.1	0.8
	R4me2	0.2	
H2B	Total (%)	15.9	16.0
	Unmodified	11.6	11.4
	P1ac	1.0	3.1
	M1acK7acK13acK14acK17acK18ac K22acK25ac	1.4	1.0
	S14p		0.2
	S14pK108ac	0.7	
	K23ac	0.9	0.4
	K108ac	0.2	
H3	Total (%)	2.8	2.6
	T3pK23acK27me2K36me2	0.1	
	K4acR8me2K9acK18acK23acK27me3K36me3	0.1	
	R8me2K9meK18acK23acK27acK36ac	0.2	
	K9meK18acK23acK27acK36ac	0.4	0.2
	K9meK18meK27me2K36ac	0.3	
	K9me2K18acK23acK27me3K36me3	0.1	0.4
	K9me2K18meK23acK27acK36ac	0.1	0.5
	K9me2K23acK27me3K36me3	0.1	
	K9me3K23meK27me3K36me3	0.1	0.2
	K9me2K27me2	0.1	
	K9meK27meK36me	0.5	0.2
	K18acK23acK27acK36ac		0.3
	K18acK23meK27me3	0.3	
	K18acK23meK27me3K36me	0.3	
	K18meK23meK27me3K36me3		0.4
	K18acK27me2K36me		0.2
	K23acK27acK36ac	0.1	
	K27me3K36me3		0.4
H4	Total (%)	56.1	25.6
	S1ac	2.3	3.0
	S1acK12acK16ac	0.2	
	S1acK12acK16acK20me	0.3	0.2
	S1acK16ac	0.1	
	S1acK16acK20me	5.7	2.1
	S1acK20me	19.4	8.3
	S1acR3meK20me	10.0	
	K5acK20me	1.6	3.0
	R3me2K12acK20me3		1.7
	R3me2K16acK20me	1.1	2.2
	R3meK20me3	5.5	5.1
	R3me2K5acK8acK20me2	4.6	
	K12acK16acK20me	5.3	

Values of total intensities normalized to 100. Underline indicates mono, di, and tri-methylation were combined.
